# Supplementary figures and images for: Fibrates ameliorate the course of bacterial sepsis by promoting neutrophil recruitment via CXCR2
Source: EMBO Mol Med. 2014 Apr 22;6(6):810–20. doi: 10.1002/emmm.201303415 (PMC4203357; doi:10.1002/emmm.201303415)

# Figure S1

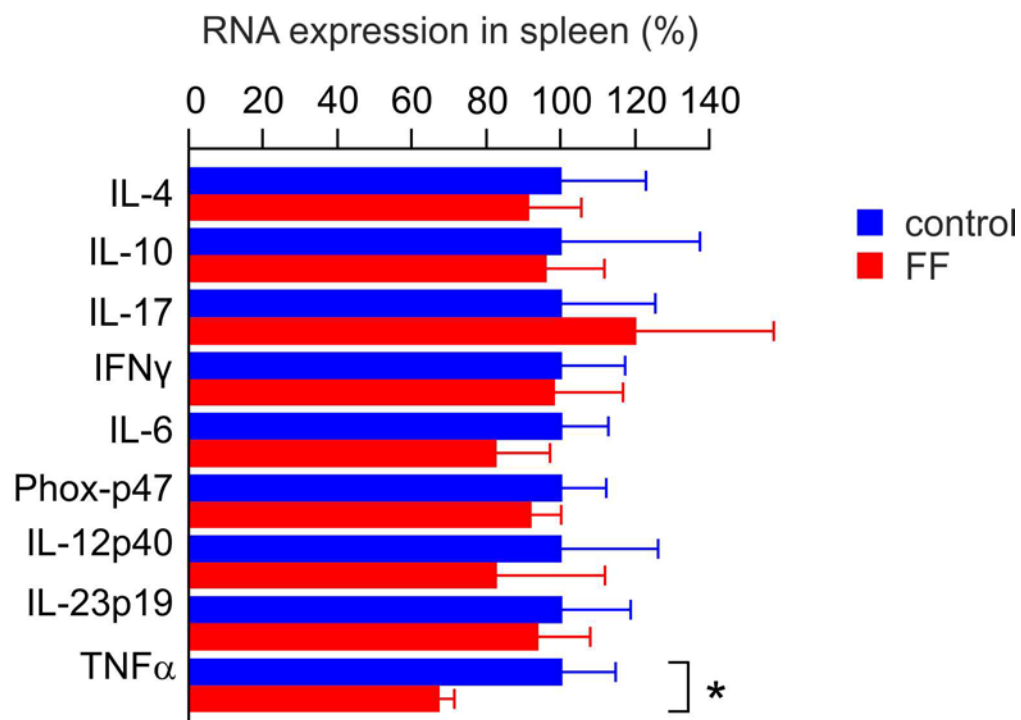

Supplement: Supplementary file 1 — Supplementary Figure S1 [file emmm0006-0810-sd1.pdf]

**Figure S2**

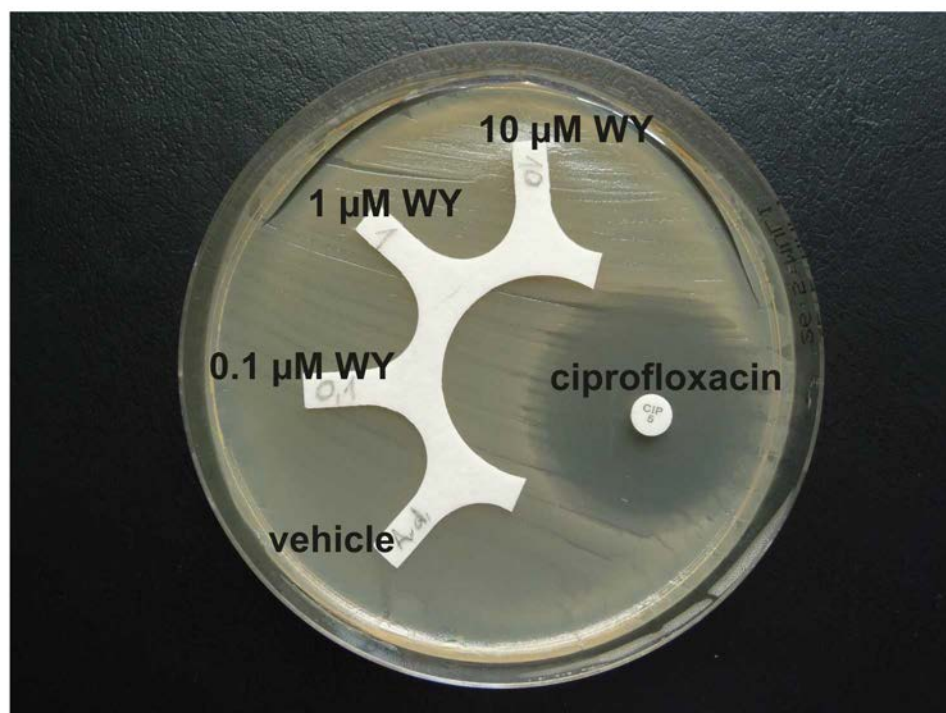

Supplement: Supplementary file 2 — Supplementary Figure S2 [file emmm0006-0810-sd2.pdf]

**Figure S3**

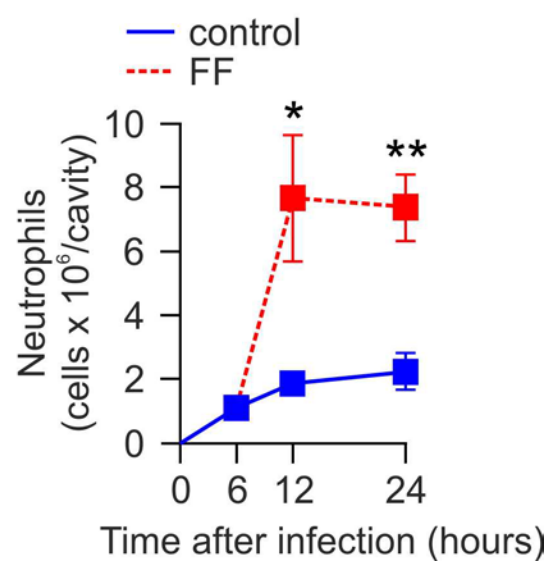

Supplement: Supplementary file 3 — Supplementary Figure S3 [file emmm0006-0810-sd3.pdf]

# Figure S4

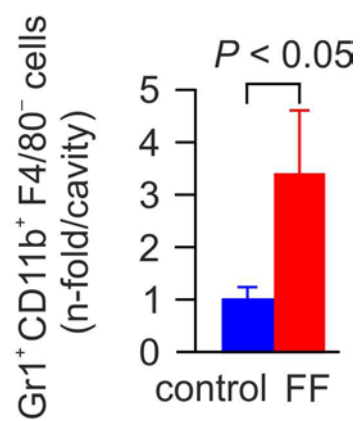

Supplement: Supplementary file 4 — Supplementary Figure S4 [file emmm0006-0810-sd4.pdf]

# Figure S5

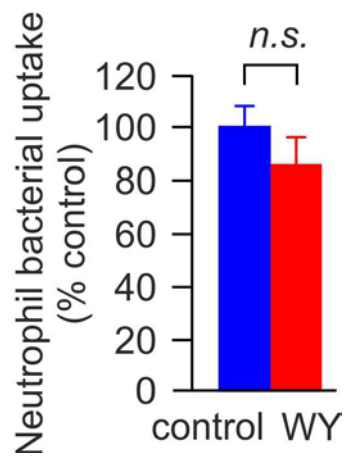

Supplement: Supplementary file 5 — Supplementary Figure S5 [file emmm0006-0810-sd5.pdf]

**Figure S6**

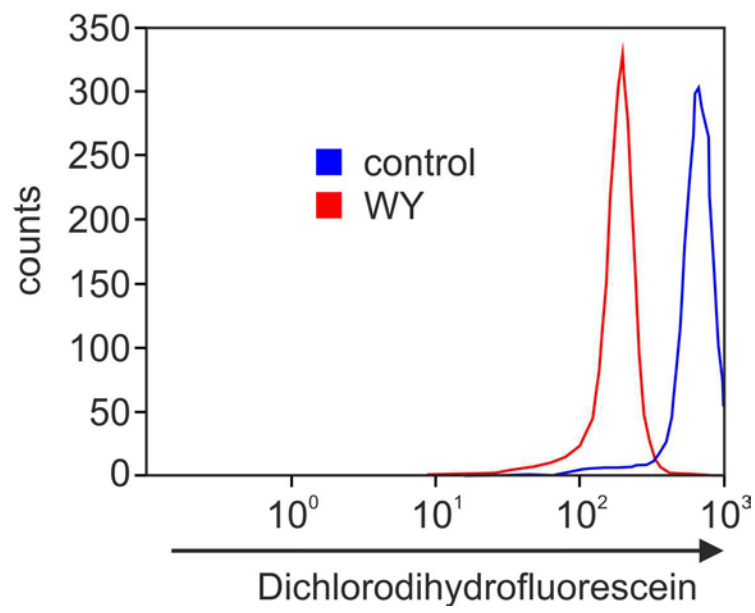

Supplement: Supplementary file 6 — Supplementary Figure S6 [file emmm0006-0810-sd6.pdf]

**Figure S7**

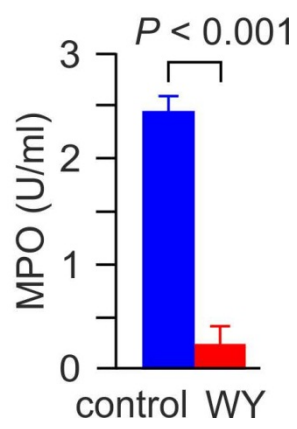

Supplement: Supplementary file 7 — Supplementary Figure S7 [file emmm0006-0810-sd7.pdf]

**Figure S8**

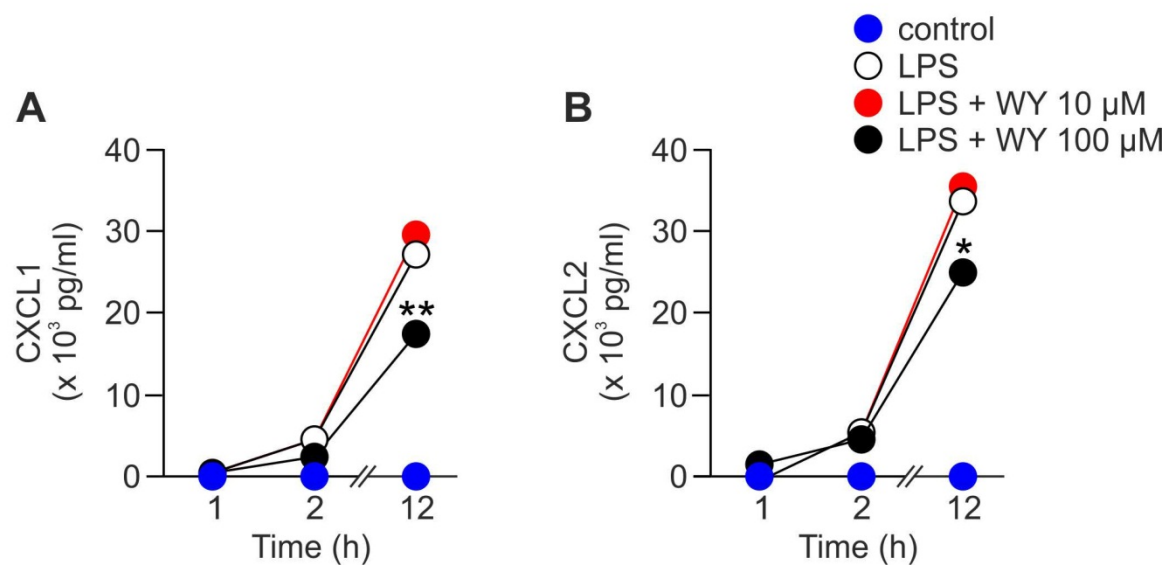

Supplement: Supplementary file 8 — Supplementary Figure S8 [file emmm0006-0810-sd8.pdf]

**Figure S9**

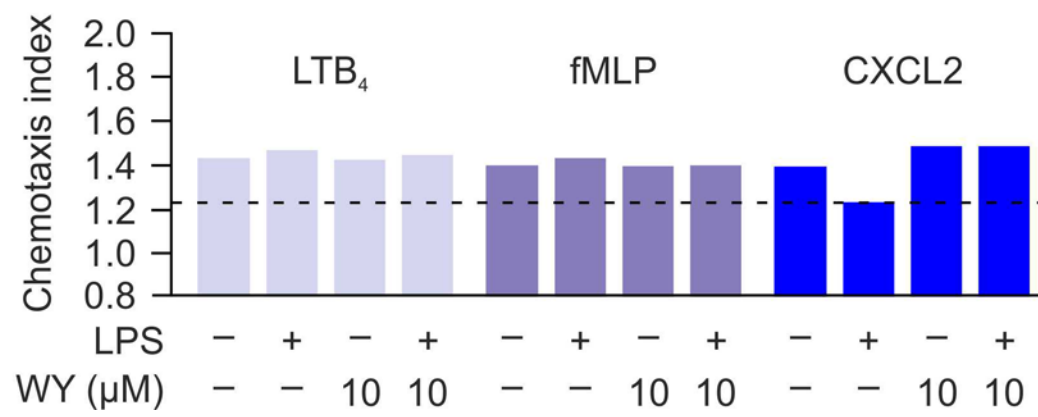

Supplement: Supplementary file 9 — Supplementary Figure S9 [file emmm0006-0810-sd9.pdf]

**Figure S10**

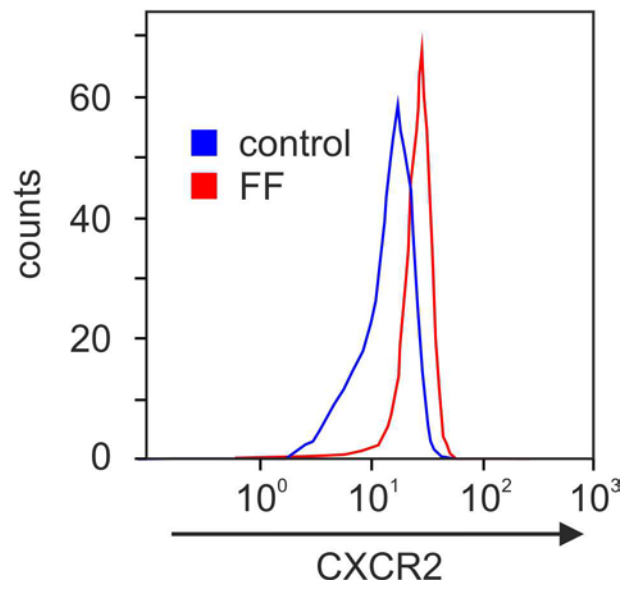

Supplement: Supplementary file 10 — Supplementary Figure S10 [file emmm0006-0810-sd10.pdf]

**Figure S11**

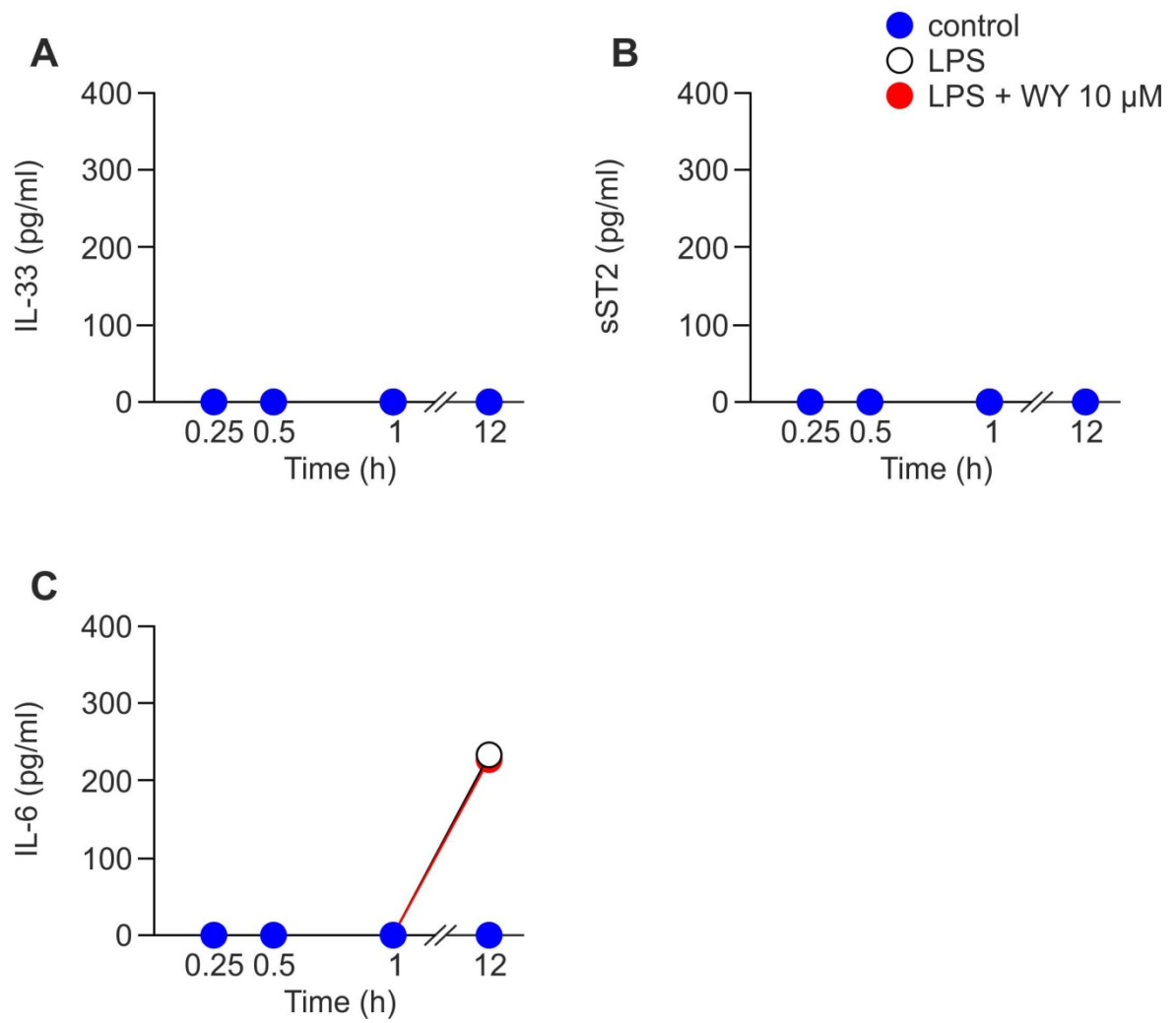

Supplement: Supplementary file 11 — Supplementary Figure S11 [file emmm0006-0810-sd11.pdf]

# Figure S12

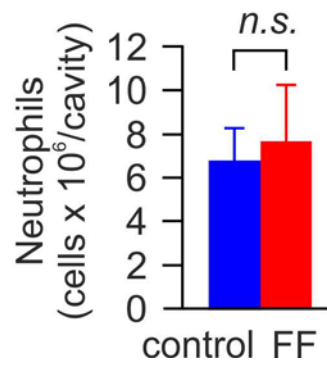

Supplement: Supplementary file 12 — Supplementary Figure S12 [file emmm0006-0810-sd12.pdf]

**Figure S13**

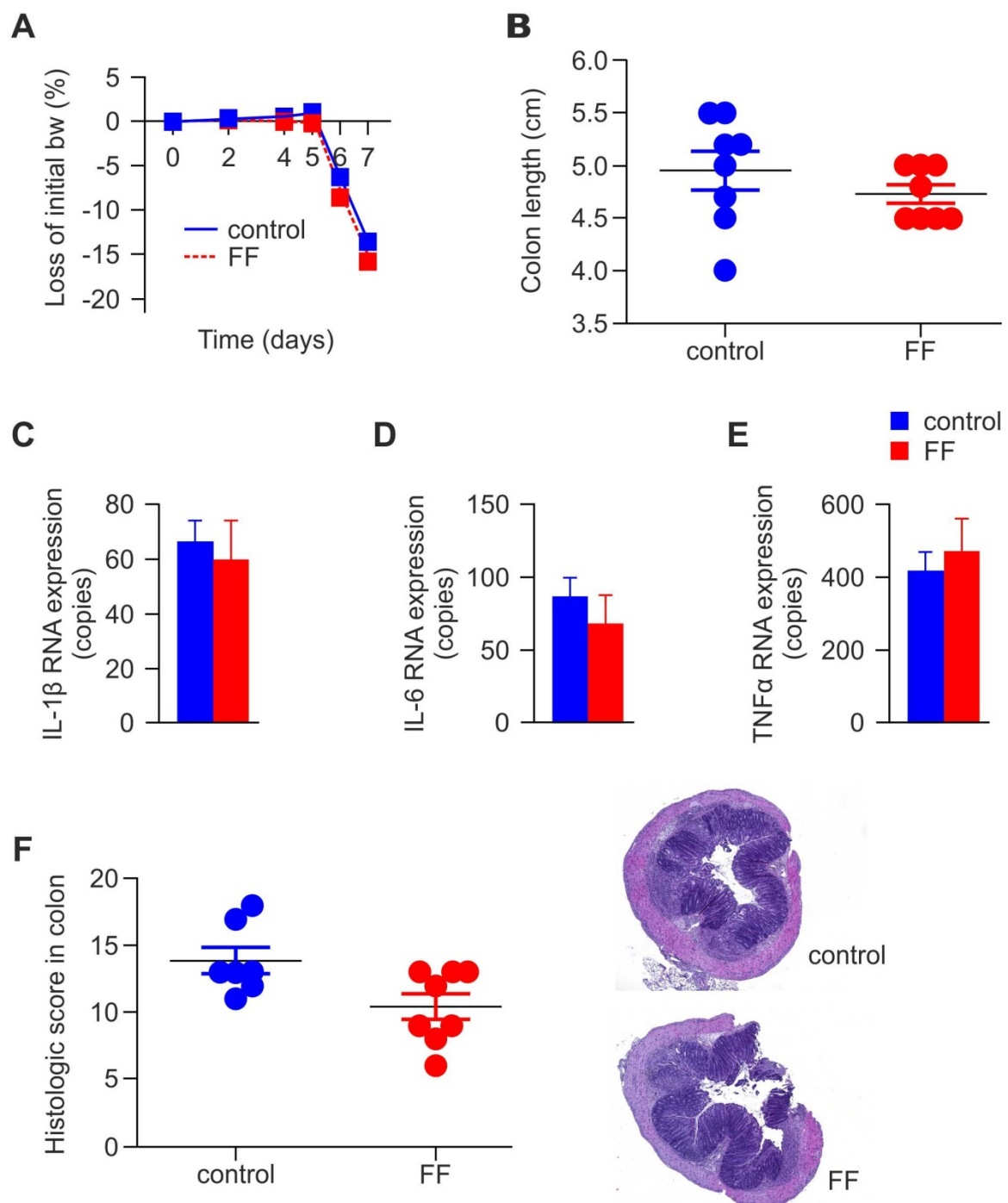

Supplement: Supplementary file 13 — Supplementary Figure S13 [file emmm0006-0810-sd13.pdf]
