# Supplementary material for: Fibrates ameliorate the course of bacterial sepsis by promoting neutrophil recruitment via CXCR2
Source: EMBO Mol Med. 2014 Apr 22;6(6):810–20. doi: 10.1002/emmm.201303415 (PMC4203357; doi:10.1002/emmm.201303415)
Supplement: Supplementary file 14 — Supplementary Figure Legends [file emmm0006-0810-sd14.pdf]

FIGURE LEGENDS

Figure S1. Effect of fenofibrate treatment on systemic inflammatory response in LPS-driven inflammation.

Mice were injected intraperitoneally with *S. typhimurium* LPS (2 mg/kg), and set on control or 0.2% FF-supplemented diet immediately after injection of LPS. 72 h later the expression of cytokines was determined in spleens of control and 0.2% FF-treated C57BL/6 mice by means of Taqman real-time PCR. Data are means ± SEM, n=6; **P*<0.05 *versus* control.

Figure S2. Fibrates do not have bactericidal activity.

Direct bactericidal activity of fibrates (WY-14643, WY at indicated concentrations) was tested in a disk diffusion test with *S. typhimurium*; ciprofloxacin served as positive control.

Figure S3. Time-course experiment with intraperitoneal determination of neutrophils. Septicemia was induced in C57BL/6 mice by intraperitoneal injection of 500 CFU of *S. typhimurium*, and the animals were set on control or 0.2% FF-supplemented chow immediately after injection of bacteria. At indicated time-points, intraperitoneal neutrophils were counted with the XE 2100TM cell counter. Data are means ± SEM, n=7; **P*<0.05, ***P*<0.01 *versus* control.

Figure S4. Intraperitoneal determination of neutrophils using FACS analysis.

Septicemia was induced in C57BL/6 mice by intraperitoneal injection of 500 CFU of *S. typhimurium*, and the animals were set on control or 0.2% FF-supplemented chow immediately after injection of bacteria. After 12 h, intraperitoneal neutrophils identified as $Gr1^+$ $CD11b^+$ $F4/80^-$ were determined. Data are means ± SEM, n=7.

Figure S5. Fibrates do not affect neutrophil bacterial uptake.

Freshly isolated murine Neutrophils were incubated with vehicle (DMSO) or the fibrate WY-14643 (WY, 10 µM) for 30 min at 37°C. Then neutrophils were infected with *S. typhimurium* at a MOI of 100 for 30 min at 37°C. Infected cells were washed with gentamicin to kill extracellular bacteria and

harvested in 0.5% sodium deoxycholic acid. The lysates were plated onto LB agar plates and incubated at 37°C. CFUs were determined after 24 h. Data are means ± SEM, n=7.

Figure S6. Fibrates decrease neutrophil ROS activity.

Reactive oxygen species (ROS) production was determined by flow cytometry, using 2’, 7’-Dichlorofluorescin diacetate. Freshly isolated murine neutrophils were pre-stimulated with vehicle (DMSO) or with the fibrate WY (100 µM) for 30 min at 37°C, and then incubated with heat-inactivated *S. typhimurium* at a MOI of 10 for 15 min. Results are expressed as mean fluorescence intensity, the histogram shows means for control and WY-treated cells, n=5.

Figure S7. Fibrates decrease neutrophil MPO activity.

Myeloperoxidase (MPO) activity was measured in freshly isolated murine neutrophils pre-stimulated with vehicle (DMSO) or with the fibrate WY (100 µM) for 30 min at 37°C. Data are means ± SEM, n=6.

Figure S8. Fibrates decrease the release of CXCL1 (A) and CXCL2 (B) from primary murine peritoneal macrophages.

Macrophages were cultured with vehicle, with LPS (1 µg/ml), or a combination of LPS with the fibrate WY-14643 (WY, 10 µM, and 100 µM) for the indicated time-points. Data are means ± SEM, n=3 per group and time-point; **P*=0.08, and ***P*<0.01 *versus* LPS at the indicated time-point.

Figure S9. Fibrates selectively improve CXCR2-mediated chemotaxis of neutrophils.

Chemotaxis to LTB_4 (100 nM), fMLP (100 nM) and CXCL2 (30 ng/ml) was determined in C57BL/6 bone marrow neutrophils pre-treated for 1 h with WY (10 µM), or in combination with LPS (1 µg/ml), as indicated. Data are pooled from 2 independent experiments.

Figure S10. Fibrates inhibit the downregulation of CXCR2 on neutrophils *in vivo*.

The expression of CXCR2 was determined by FACS analysis in blood neutrophils of *Salmonella*-infected control or 0.2% FF-treated C57BL/6 mice 6 h after intraperitoneal inoculation with the pathogen. The histogram shows mean CXCR2 expression, n=5.

Figure S11. Neutrophils do not release significant amounts of IL-33 or sST2 upon stimulation with LPS and/or fibrates.

Freshly isolated murine neutrophils were incubated with vehicle, with LPS (1 μg/ml), or a combination of LPS and the fibrate WY-14643 (WY, 10 µM) for 15, 30, 60 minutes and 12 h. **A**, IL-33 and (**B**) sST2 in cell culture supernatants were measured by ELISA. **C**, IL-6 measured by ELISA served to verify appropriate LPS stimulation of the cells. Data are means ± SEM, n=5 per group and time-point.

Figure S12. Fibrates do not affect neutrophil migration in thioglycollate-induced peritonitis.

Sterile inflammation was induced in C57BL/6 mice by i.p. injection of 1 ml 4%-thioglycollate. After 12 h, number of intraperitoneal neutrophils was determined with the XE 2100TM cell counter (n=5). Data are means ± SEM, *n.s.*= non-significant.

Figure S13. Fibrates do not influence the course of DSS-colitis.

Male C57BL/6 mice treated with 0.2%-enriched FF or control chow were administered 3% DSS dissolved in water for 5 consecutive days, which was then replaced with normal drinking water. All animals were followed up for a total of 7 days. (A) Changes in body weight (bw) and (B) colon length; (C-E) qRT-PCR analysis of immune response genes in colons of these mice, and (F) histopathological colitis scores with each point representing an individual mouse (n=8). Data are means ± SEM.
